# Identification and Expression Characteristics of the Cryptochrome Gene Family in *Chimonobambusa sichuanensis*

**DOI:** 10.3390/plants14111637

**Published:** 2025-05-27

**Authors:** Yining Kong, Changlai Liu, Tianshuai Li, Ji Fang, Guohua Liu

**Affiliations:** 1 National Key Laboratory for the Development and Utilization of Forest Food Resources, Co-Innovation Centre for Sustainable Forestry in Southern China, Bamboo Research Institute, Nanjing Forestry University, Nanjing 210037, China; kongyining2001@126.com (Y.K.); lcl2012@njfu.edu.cn (C.L.); lts123@njfu.edu.cn (T.L.); 2 Jiangsu Vocational College of Agriculture and Forestry, Nanjing 210037, China; jifang625@126.com

**Keywords:** *CRY* gene family, *Ch.sichuanensis*, blue-light stress, low-temperature stress

## Abstract

Cryptochrome is an important class of blue-light receptors involved in various physiological activities such as photomorphogenesis and abiotic stress regulation in plants. In order to investigate the molecular mechanism of blue-light-induced color change in *Chimonobambusa sichuanensis*, we screened and cloned the gene encoding the blue-light receptor Cryptochrome. In order to investigate the molecular mechanism of blue-light-induced color change in *Chimonobambusa sichuanensis*, we screened and cloned the gene encoding the blue-light receptor Cryptochrome in *Ch.sichuanensis*, and analyzed the expression characteristics of the Cryptochrome gene in *Ch.sichuanensis* under different light intensities, light quality, and temperatures by qRT-PCR. Through homologous cloning, a total of four *CsCRY* genes were obtained in the *Ch.sichuanensis* genome, namely, *CsCRY1a*, *CsCRY1b*, *CsCRY2*, and *CsCRY3*. Structural domain analyses of the encoded proteins of the four genes revealed that all CsCRYs proteins had the typical photoreceptor structural domain, PRK (protein kinase C-related kinase). Phylogenetic tree analyses revealed that the four genes *CsCRY1a*, *CsCRY1b*, *CsCRY2*, and *CsCRY3* could be categorized into three subfamilies, with *CsCRY1a* and *CsCRY1b* clustered in subfamily I, *CsCRY2* classified in subfamily II, and *CsCRY3* belonging to subfamily III. All CsCRYs proteins lacked signal peptides and the instability index was higher than 40, among which the isoelectric points of *CsCRY1a*, *CsCRY1b*, and *CsCRY2* were around five. qRT-PCR analysis revealed that the expression of all four *CsCRYs* genes was up-regulated at 75 µmol·m^−2^·s^−1^ blue-light illumination for 4 h. In addition, under treatments of different light quality, the expression of *CsCRY2* genes was significantly higher under blue light than under red light and a mixture of red light and blue light with a light intensity of 1:1; the expression of *CsCRY1a* and *CsCSY1b* was significantly higher in the mixed light of red and blue light than in the single light treatment, while under different temperature gradients, *CsCRYs* genes were highly expressed under low-temperature stress at −5 °C and 0 °C. This study provides a basis for further research on blue-light-induced color change in *Ch.sichuanensis* and expands the scope of Cryptochrome gene research.

## 1. Introduction

Bambusoideae is the largest of the 12 subfamilies in Poaceae, which currently has 127 genera and over 1680 species worldwide, including both herbaceous and woody bamboos. Among these, the genus *Chimonobambusa*, belonging to the Arundinarieae tribe and Shlbataeinae subtribe within Bambusoideae, represents an important bamboo species. It has applications in food, pharmaceuticals, timber, chemical raw materials, and landscaping, and is primarily distributed in high-altitude regions of China, Laos, Vietnam, Myanmar, and India, exhibiting strong cold tolerance [1]. The *Chimonobambusa* genera produce shoots in autumn and overwinter as shoots or young plants; their culms turn purple before winter. In our previous research on *Chimonobambusa*, specifically *Chimonobambusa sichuanensis* (commonly known as “Yueyue Bamboo”), we discovered that the color change of culms before overwintering is associated with blue light and influenced by temperature, where blue light is a necessary condition for color change, while the depth and presence of coloration are modulated by temperature. A transcriptomic analysis of *Chimonobambusa sichuanensis* culms before and after blue-light-induced color changes revealed that the blue-light receptor gene *CRY* was significantly induced by blue light [2].

Cryptochromes (CRYs) are blue-light photoreceptor proteins that play pivotal roles in regulating diverse biological processes across multiple species, including plants, animals, and microorganisms. Initially identified in Arabidopsis thaliana, CRYs are evolutionarily conserved and functionally versatile, mediating light-dependent responses such as photomorphogenesis, circadian rhythm regulation, DNA repair, and stress adaptation [3]. Cryptochromes are characterized by two primary structural domains. One is the N-terminal domain, known as the photolyase homology region (PHR), which shares high homology with photolyases, enzymes that repair UV-induced DNA damage. This domain binds flavin adenine dinucleotide (FAD) as a chromophore, enabling blue-light absorption. Key residues forming the FAD-binding cavity (e.g., TGYP and LLDAD motifs) are highly conserved, as observed in *Sorghum bicolor* SbCRY1a/1b and *Arabidopsis* AtCRY1. The C-terminal domain (C-terminal extension, CTE) is variable across species and critical for signal transduction. In plants, the CTE contains conserved motifs (e.g., DAS domain) that mediate interactions with downstream signaling partners like COP1 (Constitutive Photomorphogenic 1) and SPA (Suppressor of Phytochrome A). For instance, *Arabidopsis* CRY1 recruits the SWR1 complex via interactions with SWC6 and ARP6 to regulate H2A.Z histone deposition, thereby modulating light-responsive gene expression [4]. Previous studies have found that plants contain three main classes of Cryptochrome proteins: CRY1, CRY2, and CRY3 [5]. It is generally believed that CRY1 plays a dominant role in blue-light-induced photomorphogenesis, whereas CRY2 mainly regulates photoperiodicity to affect flowering time, and CRY3 plays an important role in the regulation of photoperiods and biological clocks. There is an overlap in the functions of the two proteins, CRY1 and CRY2, in regulating flowering. Although CRY1, CRY2, and CRY3 are the same blue-light receptor, their structural domains are very different and they have different light-responsive functions [6,7]. CRY1 and CRY2 both have a DNA photolyase domain and FAD binding domain, which mainly contain an N-terminal photorepair enzyme homology region (PHR) and C-terminal regulatory region. But not every CRY member of all species has a complete structure. For example, studies have shown that the DASH cryptochrome, also known as CRY3, lacks a C-terminal regulatory region [8]. At the same time, studies have found that corn, wheat, Arabidopsis and rice CRY1 proteins all contain a PHR domain and CCE domain, but their CRY2 proteins only have one PHR domain.

Studies have confirmed that *CRY* is involved in the response of plants to light stress. Different *CRYs* may mediate different blue-light responses through different modes of action. In Arabidopsis thaliana, *CRY1* mediates the blue-light-dependent inhibition of hypocotyl elongation, and *CRY1* can promote blue-light-induced anthocyanin accumulation. The function of *CRY2* in the early photomorphogenesis of Arabidopsis thaliana seedlings was mainly under low and medium light intensity, and the *CRY2* mutant showed an obvious hypocotyl phenotype under low-intensity blue light (<10 µmol·m^−2^·s^−1^), but did not change significantly under high-intensity blue-light stress. In addition, blue light and *CRY1* and *CRY2* were found to jointly regulate the transcription of SIG5, a transcription factor in the chloroplast signaling pathway in response to light stress in Arabidopsis [9]. Found in ferns, *CRY3* is a photoreceptor that mediates the blue-light inhibition of spore germination and plays a role in promoting the growth of plants under conditions of weak light stress.

At the same time, CRY is involved in the response of plants to temperature stress. Previous studies have found that CRY interacts with PIF4 and PIF5 in a blue-light-dependent manner. CRY1 and PIF4 interact to repress the transcription of PIF4, which in turn represses the expression of auxin-related genes such as YUC8, IAA19, IAA29, and heat-induced hypocotyl elongation by inhibiting cell elongation. CRY1 and CRY2, as well as PIF4 and PIF5, can regulate the expression of downstream genes responding to cold stress by binding to the same promoter regions of downstream genes [10]. Key genes in the CRY2 signaling pathway, such as HY5 and COP1, are involved in the regulation of cold-stress signaling. It was also found, in Arabidopsis thaliana, that CRY2 plays a role in the regulation of flowering dependent on environmental temperature, and the late-flowering phenotype of CRY2 mutants is more obvious at 16 °C than at 23 °C; that is, CRY2 mutations may lead to plants that are more sensitive to changes in environmental temperature. The temperature-responsive phenotype of the CRY2 mutant was significantly inhibited by a mutant that lost temperature sensitivity, TFL-1. But so far, there are few studies on the relationship between CRY and temperature stress. In order to explore the relationship between low-temperature stress and temperature stress in more detail, research is urgently needed. In this study, the *CsCRYs* gene family in Sichuan Province was identified and analyzed, which provided a theoretical reference for the subsequent gene cloning and functional analysis of the *CsCRYs* gene family in Sichuan Province. Although previous researchers have studied *CRY* genes in a variety of plants, there is still a lot of room for development in genetic studies of numerous bamboo species.

## 2. Materials and Methods

### 2.1. Chimonobambusa sichuanensis CsCRYs Gene Selected Based on Phyllostachys Edulis CRY Sequences

Use the protein sequence and DNA sequence of the cryptochrome gene of *Phyllostachys edulis* to search the sequence database of *Chimonobambusa sichuanensis* constructed by our laboratory [11], including the protein sequences and cDNA sequences contained in the gene database and the transcriptome database. The software used is the blast software package in TBtools v2.154, with the parameters of E-value < 1 × 10^−5^, percentage identity > 90%, and query cover > 90%, and at the same time, sequence homology analysis was performed using SnapGene v6.0.2 for multiple sequence alignment, followed by redundancy removal via BLASTN against the NCBI nr database (https://www.ncbi.nlm.nih.gov/cdd/) (accessed on 20 September 2023) (E-value set to 0.001, other parameters set to default). Four non-redundant gene sequences were retained according to their closest orthologs in the UniProtKB and Swiss-Prot databases. The portion of the *Ch.sichuanensis* Cryptochrome gene family protein sequences with high sequence similarity was presented as a visualization plot using the program ESPript 3 (https://espript.ibcp.fr/ESPript/ESPript/index.php) (accessed on 3 February 2024). The sequence similarities depiction parameter was set to PAM250, the Global score was set to 0.2 and the Diff. score was set to 0.5.

### 2.2. Structural Domain Analysis of CsCRYs

The conserved motifs of *Ch.sichuanensis CRY* protein sequences were identified using the online analysis tool MEME v5.5.8 (http://meme-suite.org/) (accessed on 25 September 2023). Parameters were set to a maximum of 10 motifs, motif lengths were set to 20–50 amino acid residues, and site distributions were set to either zero or one occurrence per sequence (contributing motif sites). At the same time, the protein sequences of 55 *CRYS* genes from other 11 species were analyzed by motif analysis, and the differences between the CsCRY protein motif of *Ch.sichuanensis* and CsCRY protein motif of other species were aligned. Using NCBI batch CD search (https://www.ncbi.nlm.nih.gov/Structure/bwrpsb/bwrpsb.cgi) (accessed on 20 September 2023), the conserved domain of the gene was obtained. The results of CDD were input by TBtools software, and the conserved domains of the *Ch.Sichuanensis CsCRYs* family were visualized. Other plant types included the following: *Arabidopsis thaliana* (ID: 826470, 839529, and 832554), *O. sativa* (LOC9270575, LOC4336008, LOC4329738, LOC9270575, and LOC4341749), *Triticum aestivum* (LOC119322215, LOC123157423, LOC123164714, LOC119317171, and LOC123149308), *Zea mays* (LOC100502533, LOC100194126, LOC133921908, and LOC100384475), *Glycine max* (LOC100797839, LOC114409283, LOC100815428, LOC100786551, LOC100783312, and LOC100233234), *Hordeum vulgare* (LOC123413527, LOC123406235, and LOC114377802), *Sorghum bicolor* (LOC110435145, LOC110436666, LOC8069379, and LOC8078292), *Populus trichocarpa* (LOC7495156, LOC7486597, LOC18100781, and LOC18102473), *Gossypium hirsutum* (LOC107957756, LOC121217897, LOC107940541, LOC107936243, LOC107905111, LOC107900827, LOC107902007, LOC107961766, and LOC107929100), *Physcomitrella patens* (LOC112284608, LOC112273862, and LOC112284678), and *Phyllostachys edulis*. The gene numbers of the alignment sequences listed above are in parentheses. A phylogenetic analysis was conducted of the *CsCRYs* gene family in *Ch.sichuanensis.*

The sequences of the plants were obtained from the TAIR10 database (http://www.Arabidopsis.org/) (accessed on 20 September 2023), Ensembe Plant database (http://plants.ensembl.org/index.html) (accessed on 20 September 2023), and PFAM database (http://pfam,sanger.ac.uk) (accessed on 20 September 2023). Mega X software v11 was used to perform multiple alignment analysis of *Arabidopsis thaliana AtCRY*, *O. sativa OsCRY*, *Sorghum bicolor SbCRY*, *Triticum aestivum TaCRY*, *Hordeum vulgare HvCRY*, *Populus trichocarpa PtCRY*, *Zea* mays *ZmCRY*, *Glycine max GmCRY*, *Gossypium hirsutum GhCRY*, *Physcomitrium sphaericum PsCRY*, *Phyllostachys edulis PheCRY*, and the conserved domain sequences of *CRY* in *Ch.sichuanensis*. Bootstrap was set to 1000 and the online software Evolview (https://www.evolgenius.info/) (accessed on 21 September 2023) was used to depict the evolutionary tree. The parameters were set to Neighbor-Join and a Bootstrap of 1000.

### 2.3. Chimonobambusa sichuanensis CsCRYs Gene Family Proteins

Subcellular localization prediction was performed using the Plant-mPLoc online tool (http://www.csbio.sjtu.edu.cn/bioinf/plant-multi/) (accessed on 2 March 2024) and the WoLF PSORT: Protein Subcellular Localization Prediction (https://wolfpsort.hgc.jp/) (accessed on 2 March 2024).

The secondary structure of CsCRYs proteins was predicted by the online tool SOPMA (https://npsa.lyon.inserm.fr/cgi-bin/npsa_automat.pl?page=/NPSA/npsa_sopma.html) (accessed on 28 April 2024). In addition, the tertiary structure of CsCRYs proteins was modeled and displayed by the Swiss-Model interactive tool (https://swissmodel.expasy.org/) (accessed on 2 March 2024). BUSCA (https://busca.biocomp.unibo.it/) (accessed on 2 March 2024) and ExPASy ProtParam (https://web.expasy.org/protparam/) (accessed on 2 March 2024) online software were used to predict physicochemical protein properties such as the molecular weight, theoretical isoelectric point, number of amino acids, and instability coefficient.

### 2.4. CsCRYs Gene Expression Pattern in Chimonobambusa sichuanensis Under Different Environmental Conditions

Firstly, light quality was set as the environmental variable, and *Ch.sichuanensis* hydroponic seedlings were cultivated in the greenhouse of Nanjing Forestry University Bamboo Research Institute, cultured at a constant temperature of 20 °C. Hydroponics seedlings were treated with three kinds of light without interruption: single blue light and red light with a light intensity of 100 µmol·m^−2^·s^−1^, and mixed light with a total light intensity of 100 µmol·m^−2^·s^−1^. The mixed light was composed of red light and blue light with a light intensity ratio of 1:1.

Next, light intensity was set as the environmental variable, and the bamboo seedlings were irradiated uninterruptedly using blue light with total light intensities of 50, 75, and 100 µmol·m^−2^·s^−1^. Finally, temperature was set as the environmental variable, and bamboo seedlings were irradiated using constant natural light with a light intensity of 100 µmol·m^−2^·s^−1^, and treated with different temperature gradients: −5 °C, 0 °C, 5 °C, 10 °C, and 20 °C. Three pots of bamboo seedlings with similar growth were set as the replicate control, and leaves were collected every 4 h. The sampling times were 0 h, 4 h, 8 h, 12 h, 16 h, 20 h, and 24 h. First, the RNA extraction kit from Vazyme Biotech company (Nanjing, China), the VeZol-Pure Total RNA Isolation Kit, was used and sample RNA was extracted. Secondly, the cDNA: HiScript IV 1st Strand cDNA Synthesis Kit (+gDNA wiper) and reverse transcription reagent were obtained from Vazyme Biotech company. Finally, a quantitative reagent from Hunan Accurate Biology company, the SYBR greenpro Taq HS plant qRT-PCR kit, was used to quantitatively detect the expression of cryptochrome *CsCRYs* in the leaves of *Ch.sichuanensis* in the different environments by qRT-PCR. The quantitative primers and PCR amplification procedures are shown in Table 1, and the spectra of processing conditions are shown in Figure S2 in the Supplementary Materials.

## 3. Results and Analysis

### 3.1. Sequence Domain of CsCRYs Protein in Chimonobambusa sichuanensis

**Figure 1:**
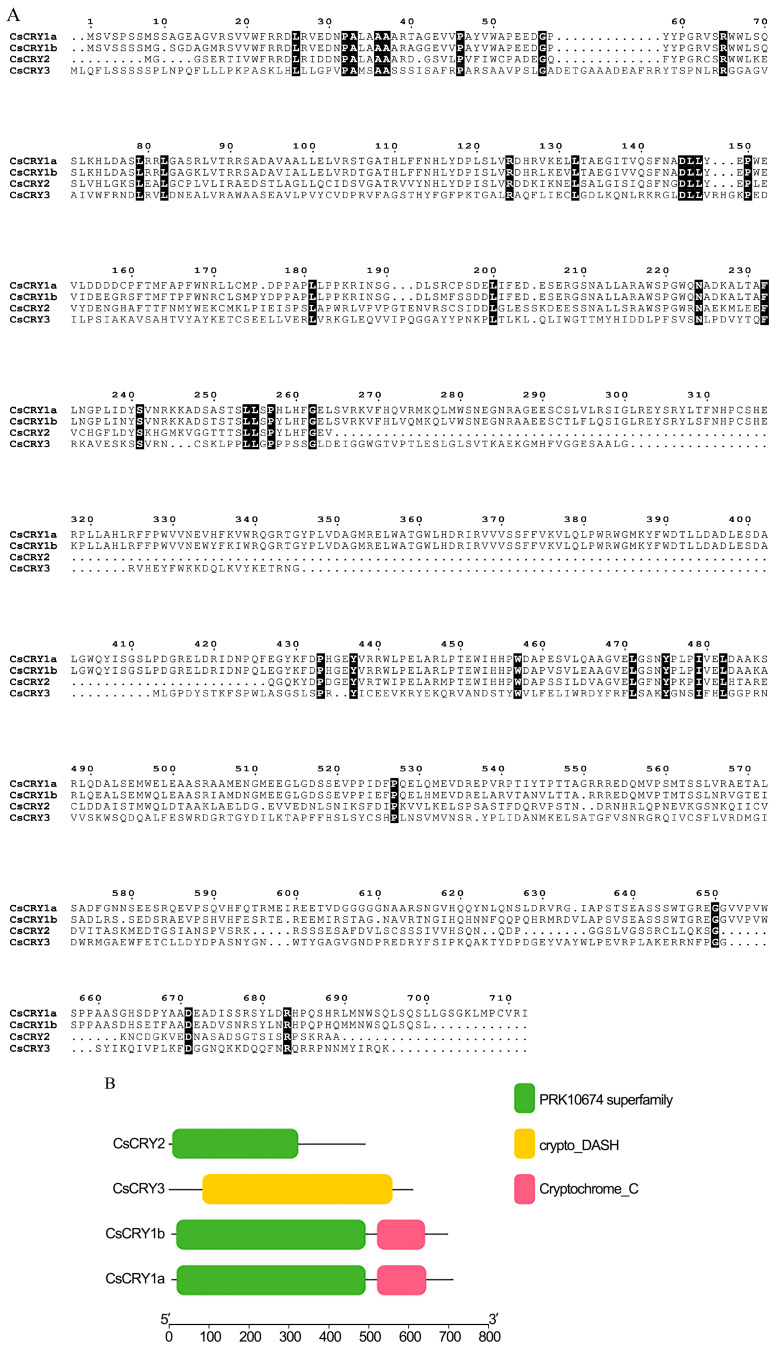
Sequence alignment and analysis of *Ch.sichuanensis CsCRYs* gene family proteins. Note: (**A**) Protein sequence alignment of the *Ch.sichuanensis CsCRYs* gene family; (**B**) analysis of conserved domains of the *Ch.sichuanensis CsCRYs* gene family.

Using the *Phyllostachys edulis CRY* genes as reference genes, four *CRY* gene sequences of *Ch.sichuanensis* were obtained through homology comparison. The four *CRY* sequences were aligned with the NR database to confirm their identity as *CRYs* genes. Based on the homology alignment results, the four sequences were named *CsCRY1a*, *CsCRY1b*, *CsCRY2*, and *CsCRY3*. Among them, *CsCRY1a* is 2139 bp in length and encodes 712 proteins; *CsCRY1b* is 2094 bp in length and encodes 697 proteins; *CsCRY2* is 1476 bp in length and encodes 491 amino acids; and *CsCRY3* is 1833 bp in length and encodes 610 amino acids (Figure 1A).

Using MEGA v11 software, the sequence alignment of the four CRYs proteins was performed to construct their evolutionary relationships. *CsCRY1a* and *CsCRY1b* showed the highest similarity at 34.21% similarity, while *CsCRY2* and *CsCRY3* had a similarity of 21.48%. The four CsCRYs protein sequences share only 72 common amino acids. The structural domain analysis of the four CsCRYs proteins using ESPript 3 revealed that CsCRY1b and CsCRY1a have identical domains, both possessing the PRK10674 superfamily and Cryptochrome C domains (Figure 1B), as well as the photolyase homology region (PHR) domain and the C-terminal extension domain. CsCRY2 only contains the PRK10674 superfamily domain, while CsCRY3 only has the crypto_DASH domain, showing significant differences.

### 3.2. Structural Characterization of Chimonobambusa sichuanensis CsCRYs Gene Family

**Figure 2:**
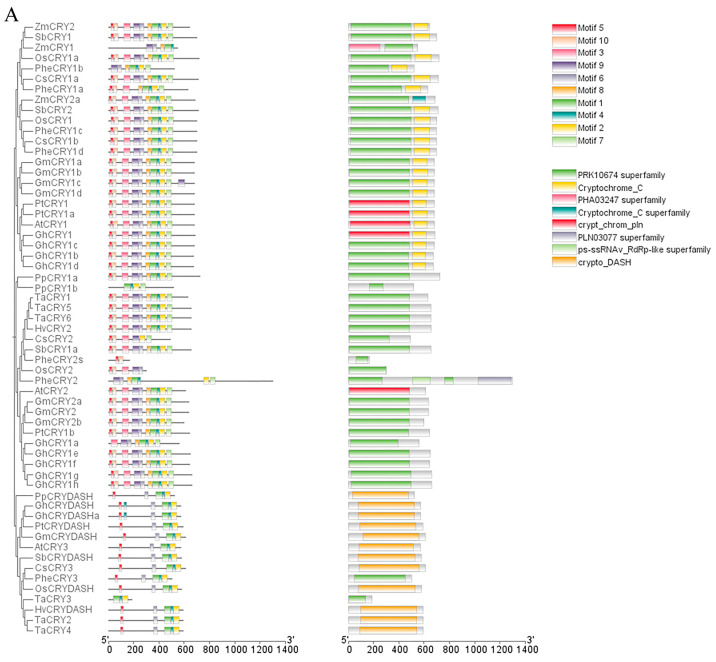

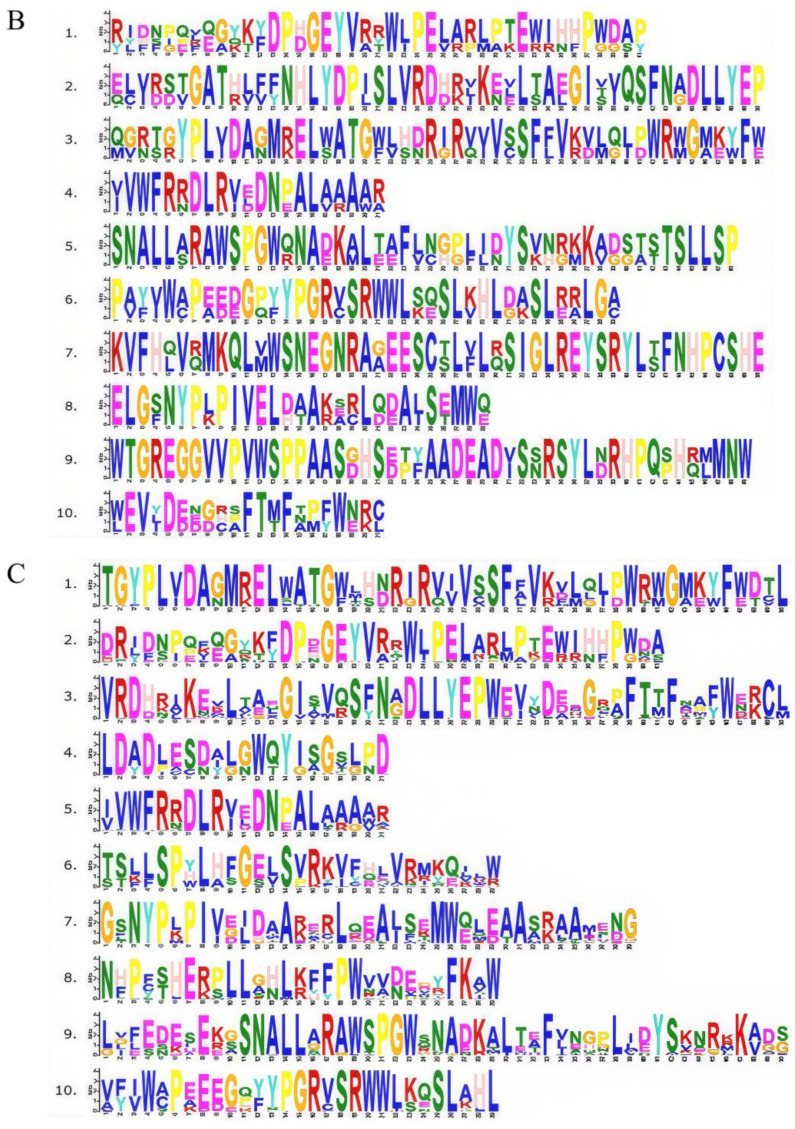
Evolutionary tree and motif comparison of *CRYs* gene families in multiple species. Note: Plant species include Chimonobambusa sichuanensis (Cs), Arabidopsis thaliana (At), O. sativa (Os), Triticum aestivum (Ta), Zea mays (Zm), Glycine max (Gm), Hordeum vulgare (Hv), Sorghum bicolor (Sb), Populus trichocarpa (Pt), Gossypium hirsutum (Gh), Physcomitrella patens (Pp), and Phyllostachys edulis (Phe). (**A**) Evolutionary tree and conserved structural domain analysis of *CRY* gene families in multiple species; (**B**) *Chimonobambusa sichuanensis CsCRYs* gene family motifs; and (**C**) *CRY* gene family motifs in multiple species.

In order to explore the structural characteristics of CsCRYs, four genetic structures were visually analyzed using MEME and TBtools, and the results are shown in Figure 2A. Motif analysis showed that Motifs 1, 4, 6, and 7 were present in the four CsCRY proteins CsCRY1a, CsCRY1b, CsCRY2, and CsCRY3, and Motifs 1, 8, and 9 together constituted the homologous domain of the CsCRY1 subfamily, as shown by the conserved domain analysis (Figure 2C). The conserved motifs of CsCRY1a and CsCRY1b are the same so that they can be integrated into the same subfamily. The presence or absence of Motif 9 is the criterion for the identification of the CsCRY1 subfamily. The CsCRY2 subfamily differs from the other subfamilies in that Motif 3 is missing. Similarly, the CsCRY3 subfamily did not contain Motifs 2, 5, 8, and 10 compared to the other subfamilies (Figure 2A). Motifs 1, 2, 4, 5, 6, 8, and 10 constitute the PRK10674 conserved domain, which is common to photoreceptors, and Motif 9 constitutes the Cryptochrome_C conserved domain, which is unique to the *CsCRYs* family. Conserved motif analysis revealed that Motif 2, Motif 4, and Motif 6 are highly conserved among all family members, indicating that these motifs play a fundamental and important role in CRY protein function (Figure 2B). The motif differences between subclasses are as follows: CsCRY1 has Motif 3 and Motif 7 compared to CsCRY2, and CsCRY3 was identified as Cryptochrome_DASH due to the lack of Motif 2 and Motif 10, whereas CsCRY1 and CsCRY2 are from different subclasses but contain Motif 5, and there is slight difference between different subclasses. 

To further explore the similarities and differences between the *CsCRYs* gene family and other species, the conserved motifs of the *CRY* gene family in the four *CsCRYs* genes and several species were compared and analyzed (Figure 2A). A total of 59 gene sequences, distinguished by 10 conserved motifs (Motif 1–Motif 10), were examined using the online software MEME for the *CRY* gene families in the four *CsCRYs* genes and other species. The results showed that each *CRY* gene contains 1 to 10 motifs. An analysis of this result in conjunction with Figure 2A,C shows that *CsCRY1a* and *CsCRY1b* have the same motif and both consist of two structural domains; Motifs 3, 5, 6, 9, and 10 form the PRK10674-Superfamily structural domain, which is related to signaling; and the rest, Motifs 1, 2, 7, and 8, form the Cryptochrome-C structural domain, which is related to photosynthetic signaling and interaction with downstream signals, and is similar to other species such as wheat, sorghum, and maize, which also consist of these two structural domains in their CRY1 sequences and have the same motif composition, which can be regarded as a strong evidence for the identification of *CRY1*. Interestingly, *Phyllostachys edulis PheCRY1a*, which is very close to *Ch.sichuanensis*, lacks Motifs 3, 5, and 10, which suggests that there are differences in their sequence compositions despite having the same structural domains, and *CsCRY2* is similar to *CRY2* genes of other species in that it possesses a PRK10674-Superfamily structural domain, which is not of the same length in different species. *CsCRY2* consists of Motifs 2, 3, 5, 7, 9, and 10, with Motif 8 missing compared to wheat, sorghum, and maize, and this fragment is not found in *Phyllostachys* edulis (Figure 2A). *CRY3* is more consistent in all species and consists of the Crypto-DASH structural domain, which is a marker for identifying CRY3 and consists of Motifs 1, 2, 4, 5, and 6, which is present in both *CsCRY3* and *AtCRY3* and *ZmCRY3*.

### 3.3. Genetic Relationship of Chimonobambusa sichuanensis CsCRYs Gene

In order to explore the genetic relationship between *CsCRYs* genes of *Chimonobambusa sichuanensis* and other species, four CsCRYs protein sequences of *Chimonobambusa sichuanensis* were aligned with 55 CRYs protein sequences of 10 other species, and the results were as shown in the phylogenetic tree in Figure 2A; all *CRYs* genes were divided into three subfamilies. The *CsCRYs* gene of *Chimonobambusa sichuanensis* was distributed in all three subtribes (subtribes I-III). Among them, there are two members in subfamily I (*CsCRY1a*, *CsCRY1b*), one member in subfamily II (*CsCRY2*), and one member in subfamily III (*CsCRY3*). The members belonging to the same subclade have the same protein structure, and the branches (*CsCRY1a~3*) are consistent with the results of the conserved motif analysis. The evolutionary relationship can be seen in that *CsCRY1a* and *CsCRY1b* are clustered into a subfamily with *PheCRY1a* and *PheCRY1b* of *Phyllostachys edulis*, and *CsCRY1a* and *PheCRY1a* are clustered into a branch in the evolutionary tree before polymerizing with *CsCRY1b*, which proves that in this subfamily, *CsCRY1a* is more closely related to *PheCRY1a* (Figure 2A). *CsCRY1a*, *CsCRY1b, PheCRY1a*, and *PheCRY1b* all have the PRK10674-Superfamily as well as Cryptochrome-C structural domains, which are shared by members of this subfamily in the evolutionary tree, which is a common feature of subfamily I. *CsCRY1a*, *CsCRY1b*, and *PheCRY1a* are all related to *PheCRY1a*. Similarly, *CsCRY2* is located in subfamily II and is closer to *PheCRY2s*, which proves that in this subfamily, *CsCRY2* is more closely related to *PheCRY2s* (Figure 2A). Both *CsCRY2* and *PheCRY2* have the PRK10674-Superfamily structural domain, which is shared by members of subfamily II in the evolutionary tree. *CsCRY3*, in turn, constitutes a small branch with *PheCRY3*, which proves that *CsCRY3* is more closely related to *Phyllostachys edulis PheCRY3* in subfamily III (Figure 2A). Both *CsCRY3* and *PheCRY3* have the Crypto-DASH structural domain, which is shared by members of this subfamily in the evolutionary tree, and is a common characteristic of subfamily III as well as a criterion for its identification. All four *CsCRYs* family sequences of *Ch.sichuanensis* showed very close affinity with the *PheCRYs* family sequences of *Phyllostachys edulis* and were highly similar in classification, which proved the correctness of the screening. The branching of the phylogenetic tree can effectively reflect the evolutionary relationship of *CRY* genes in each species.

Among them, some motifs are common to all members, such as Motif 5 (except *TaCRY3*, *PpCRY1b*, and *ZmCRY1*), and *CRY* gene family members of most species contain Motif 1, Motif 2, Motif 3, Motif 4, Motif 5, and Motif 10. The results indicate that they are *CRY* gene family members and Motifs 3, 5, 6, 9, and 10 constitute the PRK10674 conserved domain, Motifs 1, 2, 4, 7, and 8 constitute the Cryptochrome_C conserved domain, and Motifs 1, 2, 4, and 6 constitute the Cryptochrome_DASH domain (Figure 2A,B). The above results indicate that there are both similarities and differences in the functions of *CRY* genes among different subfamilies.

A total of four members of the *Ch.sichuanensis CsCRYs* gene family were accurately identified through the mining of *Ch.sichuanensis* transcriptome data, and this number was determined from seven *Phyllostachys edulis* sequences screened by previous authors, among which *PheCRY3* and *PheCRY4* were demonstrated to have repetitive sites and could be spliced into a single sequence in the present study. This is consistent with the classification of *CRY* families in other plant species such as *Arabidopsis*, rice, companion mineral Sedum, alfalfa, and soybean (Figure 2C). All four screened sequences of *Ch.sichuanensis* corresponded to *Phyllostachys edulis* sequences, which can be classified into three subfamilies (subfamilies I-III) based on their structural features. Compared with the classification results of other species, which are similar to that of *Arabidopsis*, the *Arabidopsis* cryptochrome gene family consists of members of three subfamilies: *AtCRY1*, *AtCRY2*, and *AtCRY3*. Among them, *CRY3* belongs to the CRY-DASH branch, and evolutionarily, *CRY3* is an intermediate between Cryptochrome and photolyase. In contrast, although the *PheCRY* gene family of *Phyllostachys edulis* is divided into three subfamilies, the number in each subfamily is not equally distributed, which is different from *Arabidopsis* and *Ch.sichuanensis*. By analyzing other species classifications, all of these results showed differences, implying that there are structural and functional differences in *CRY* proteins from different plant species.

### 3.4. Physicochemical Properties and Secondary Structures of Four CsCRYs Family Proteins from Chimonobambusa sichuanensis

The protein lengths of *Chimonobusa sichuanensis* CsCRYs ranged from 491 to 712 bp, and most *Ch.sichuanensis CsCRYs* genes and protein lengths had little variation, except for CsCRY2, which had the smallest protein length. In order to understand the relative molecular mass and isoelectric point of CsCRYs proteins, the physical and chemical properties of CsCRYs were analyzed by ProtParam software on the Expasy website, and the relative molecular mass of the four proteins was 54. The relative molecular mass of the CsCRY2 protein was the smallest, and the relative molecular mass of CsCRY1a was the largest. From the isoelectric point analysis of the four proteins, it can be seen that the isoelectric point of the CsCRY3 protein is greater than 7, which indicates a basic protein, and the isoelectric point of the other three CsCRYs proteins is about 5, which indicates an acidic protein. The instability coefficients of the four CsCRYs ranged from 41.41 to 54.36, all of which were greater than 40, indicating that they were unstable proteins. The average hydrophilicity coefficient of the CsCRYs protein was −0.433~−0.317, which was less than 0, so it was a hydrophilic protein. Protein location analysis showed that CsCRY1a and CsCRY1b were distributed in the cytoplasm, while CsCRY2 was located in the nucleus and CsCRY3 was located in the chloroplast (Table 1). Using Signal p6.0 (https://services.healthtech.dtu.dk/services/SignalP-6.0/) (accessed on 10 April 2024) and the TMHMM website (https://services.healthtech.dtu.dk/services/TMHMM-2.0/) (accessed on 10 April 2024) showed that the signal peptide ratio Sec/SPI of the four proteins was 0, and the signal peptide index was less than 0.5. The results showed that there was no signal peptide in the CsCRYs protein of *Ch.sichuanensis*. It can be seen from the comprehensive analysis of the results in Figure S1 in the Supplementary Materials that the CsCRYs protein of the *Ch.sichuanensis* has no signal peptide and no transmembrane domain, which is consistent with the above subcellular prediction results.

**Figure 3:**
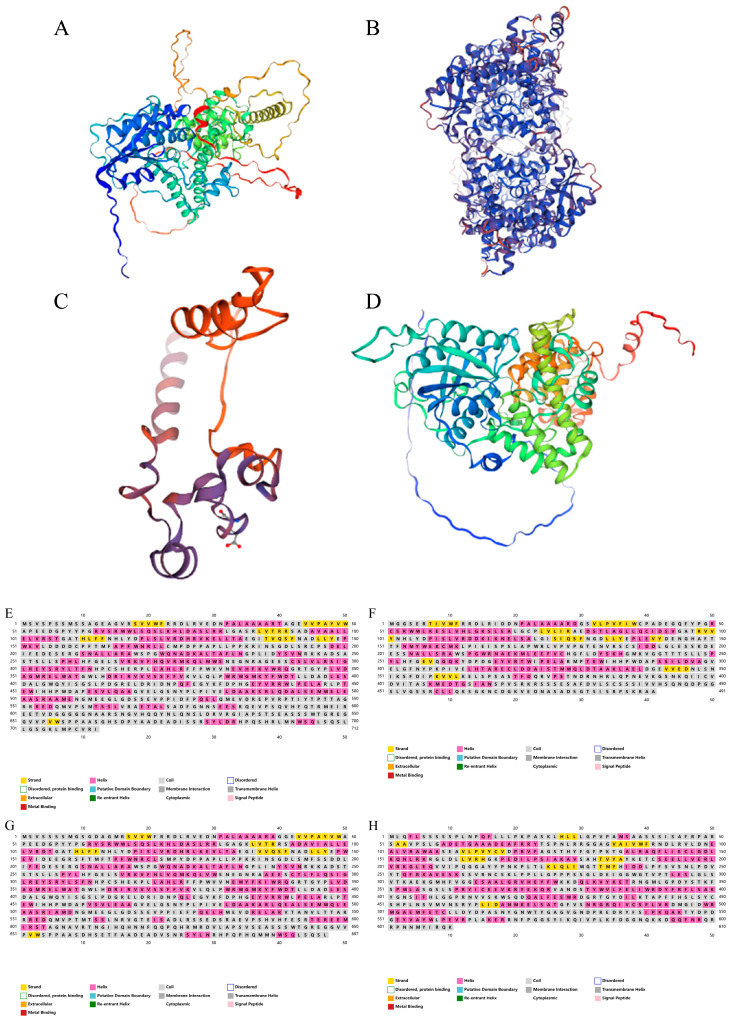
Protein structure prediction of CsCRYs family members in *Chimonobambusa sichuanensis.* Note: (**A**) Predicted secondary structure of CsCRY1a protein; (**B**) predicted secondary structure of CsCRY1b protein; (**C**): predicted secondary structure of CsCRY2 protein diagram; (**D**) CsCRY3 protein secondary structure prediction diagram; (**E**) predicted 3D structure of CsCRY1a protein, which is based on the construction of a corn CRY1 gene model with a matching similarity degree of 88.97%, gene number LOC100384475; (**F**) predicted 3D structure of CsCRY1b protein, which is based on the construction of a corn CRY1 gene model with a matching similarity of 82.01%, gene number LOC100384475; (**G**) predicted 3D structure of CsCRY2 protein, based on a maize OsCRY2 gene model construct with a matching similarity of 80.37%, number J013042O16; and (**H**) predicted 3D structure of CsCRY3 protein, which is based on the construction of the OsCRY3-DASH gene model of Japonicoideae with a matching similarity of 88.79%, number Q651U1.

In order to further analyze the molecular structure of the four proteins, the three-dimensional structure of the four CsCRYs proteins was analyzed through the database PSIPRED (http://bioinf.cs.ucl.ac.uk/psipred/) (accessed on 10 April 2024). The results showed that a random coil was the most dominant secondary structure (Figure 3, Table 2) in the four CsCRYs proteins, and the proportion of α-helix was between 34.62% and 39.17%, and the proportion of β-sheet was between 4.16% and 8.35%. The evaluation of the 3D model predictions showed low values for both Ramachandran Outliers and Clash Score, indicating a high reliability of the model predictions (Table 2).

### 3.5. Expression Pattern of Cryptochrome CsCRYs Gene in Chimonobambusa sichuanensis Under Different Environmental Condition

**Figure 4:**
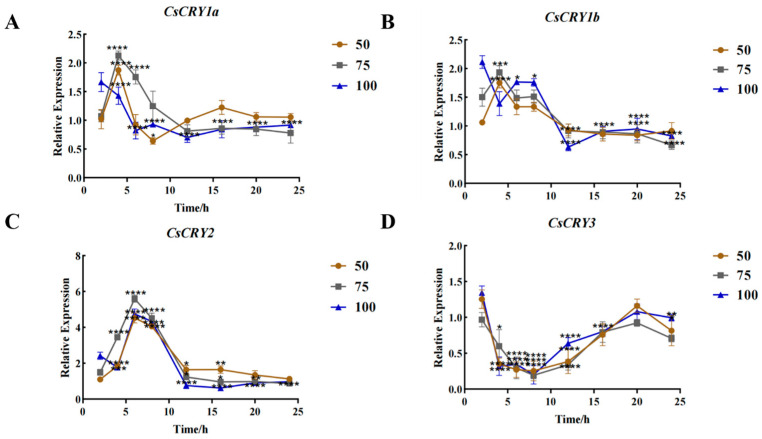
Gene expression of *Chimonobambusa sichuanensis CsCRYs* family members under different blue-light treatments. Note: (**A**) CsCRY1a gene expression; (**B**) CsCRY1b gene expression; (**C**) CsCRY2 gene expression; and (**D**) CsCRY3 gene expression. Data were normalized by qRT-PCR to the expression level of each gene at 3 h. Values are expressed as mean ± SD. Statistically significant differences were analyzed using two-way ANOVA (* *p* < 0.05, ** *p* < 0.01, *** *p* < 0.001, and **** *p* < 0.0001).

In order to analyze the functional differences of four *CsCRYs* genes in *Chimonobambusa sichuanensis*, the hydroponic seedlings of *Chimonobambusa sichuanensis* were used as experimental materials, and the gene expression levels were analyzed by setting different light intensities and treatments. The gene expression of the four *CsCRYs* genes had certain differences after being treated by different light intensities, among which *CsCRY1a*, *CsCRY1b*, and *CsCRY2* genes responded rapidly in a short period of time (2–6 h), and the gene expression of the three genes increased significantly after being treated by blue light for 2–6 h. The gene expression response (12–24 h) decreased (Figure 4A–C), while the expression pattern of *CsCRY3* gene expression showed a down–up–down trend (Figure 4D), showing special expression characteristics. The expression of *CsCRY1a*, *CsCRY1b*, and *CsCRY2* genes was the highest under 75 µmol·m^−2^·s^−1^ blue light in the rapid response stage (2–6 h) of light treatment. However, the 50 µmol·m^−2^·s^−1^ treatment group showed higher gene expression during the 12–24 h period of the blue-light treatment of these three genes. It is speculated that *CsCRY1a*, *CsCRY1b*, and *CsCRY2* are continuously adapted to low-intensity blue light. The expression of the *CsCRY3* gene in the 100 µmol·m^−2^·s^−1^ light treatment group was significantly higher than that in other groups at most time points (*p* < 0.01), and may be involved in the regulation of the long-term stress of high light intensity, and may be related to environmental adaptability. It can be seen from Figure 4 that *CsCRY1a*, *CsCRY1b*, and *CsCRY2* mediate short-term blue-light stress, and *CsCRY3* plays a unique role in sustained high light stress. Quantitative primers and internal reference genes are shown in Table S1 in Supplementary Materials, and the spectra of treatment conditions are shown in the Figure S2 in Supplementary Materials.

**Figure 5:**
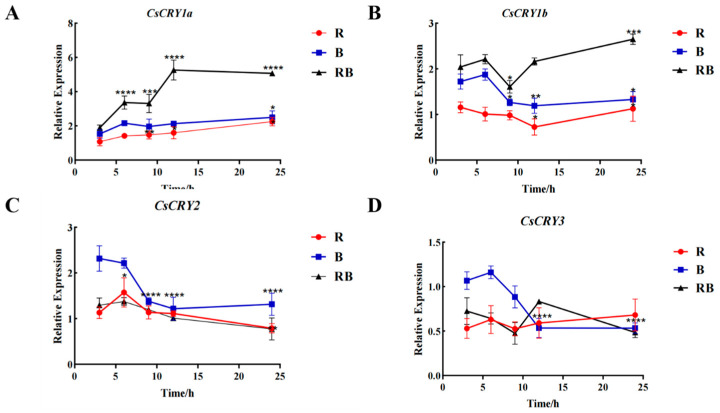
Gene expression of *Chimonobambusa sichuanensis CsCRYs* family members under different light quality treatments. Note: (**A**) *CsCRY1a* gene expression; (**B**) *CsCRY1b* gene expression; (**C**) *CsCRY2* gene expression; and (**D**) *CsCRY3* gene expression. Data were normalized by qRT-PCR to the expression level of each gene at 3 h. Values are expressed as mean ± SD. Statistically significant differences were analyzed using two-way ANOVA (* *p* < 0.05, ** *p* < 0.01, *** *p* < 0.001, and **** *p* < 0.0001).

In order to understand the response of the four genes to different light qualities, bamboo seedlings were treated with different light qualities, and the gene expression of *CsCRYs* was shown in Figure 5. The gene expression levels of both *CsCRY1a* and *CsCRY1b* were the highest under red and blue mixed light (Figure 5A,B). The response of *CsCRY2* to blue light was higher than that to red light and red–blue mixed light, and the expression of *CsCRY3* was relatively high in the early stage of blue-light treatment (2–10 H), but the response of *CsCRY3* to different color light was not significant after 10 H of different quality light treatment.

**Figure 6:**
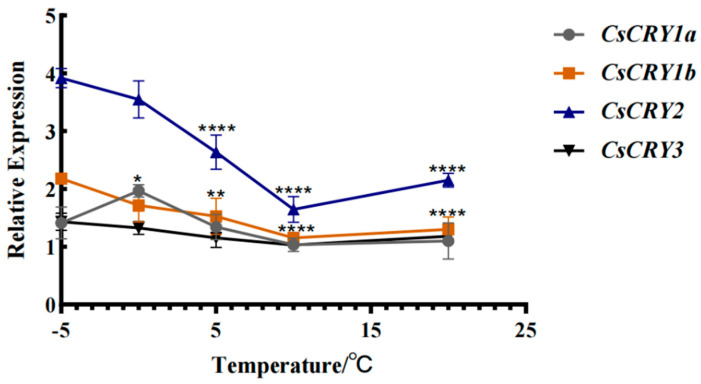
Gene expression of *Chimonobambusa sichuanensis CsCRYs* family members under different temperature treatments. Note: Data were normalized by qRT-PCR to the expression level of each gene at −5 °C. Values are expressed as mean ± SD. Statistically significant differences were analyzed using two-way ANOVA (* *p* < 0.05, ** *p* < 0.01, and **** *p* < 0.0001).

In order to explore the relationship between the *CsCRYs* gene and temperature in *Ch.sichuanensis*, bamboo seedlings were treated at different temperatures (−5 °C to −20 °C). The results showed that with the decrease in treatment temperature, the expression of *CsCRY2* increased significantly, reaching the peak at −5 °C (Figure 6), which was consistent with the data from the transcriptome study on *Ch.sichuanensis* culms in winter in our laboratory, and confirmed that it was involved in the regulation of cold resistance. The expression of other members *CsCRY1a*, *CsCRY1b,* and *CsCRY3* did not change significantly, and the expression of the three genes increased slightly with the decrease in temperature, indicating that there was functional differentiation among the *CsCRYs* gene families. The results showed that *CsCRY2* had both blue-light sensing and low-temperature response functions in *CsCRYs*, and it was inferred that *CsCRY2* was the key molecule for bamboo to adapt to a winter environment.

## 4. Discussion

### 4.1. Structural Domain Differences Drive the Functional Differentiation of CsCRYs in Chimonobambusa sichuanensis

First, this study speculated that the conserved domain is related to the short-term stress response: by analyzing the composition of the conserved motif of CsCRYs, it was found that CsCRY1a, CsCRY1b, and CsCRY2 all contain the PRK10674-Superfamily domain (Figure 2), which is highly conserved (Section 2.3) and may explain the consistency in the expression trend of the three genes in the short-term response (2–6 h) to low-to-medium-intensity blue light (50–75 µmol·m^−2^·s^−1^) (Figure 4A–C). After this conserved domain binds to FAD and FMN, it can participate in the signal transduction process and regulate the expression of downstream genes and cell activities. The difference is that in the sequences of Phyllostachys pubescens screened by predecessors, no similar PheCRY3 protein has this domain. Moreover, through domain analysis, it was found that PheCRY3 and PheCRY4, as determined in previous publications, have high repeatability, and may have the same sequence. The above results are consistent with the mechanism of early blue-light signal transduction mediated by *AtCRY1* and *AtCRY2* in *Arabidopsis thaliana* [12], suggesting that members of this subfamily may participate in stress response through photoreceptor-COP1 interaction in *Ch.sichuanensis*.

Secondly, the Crypto-DASH domain is involved in long-term stress regulation: the unique Crypto-DASH domain of CsCRY3 (Figure 2) may regulate its functional specificity by binding to FAD/MTHF. As shown in Figure 4D, CsCRY3 exhibits a high expression pattern under 100 µmol·m^−2^·s^−1^ blue light, which is presumed to be involved in long-term high light stress adaptation [13]. This phenomenon is similar to the mechanism by which AtCRY3 disrupts DNA repair in *Arabidopsis* [14], suggesting that members of the Crypto-Dash subfamily may respond to persistent environmental stress through conformational stability regulation. The structural analysis of the above four CsCRYs proteins once again confirms that the structure of the gene is closely related to its function, and the domain differences drive the functional differentiation of CsCRYs in *Ch.sichuanensis*. These results reflect the unity and variability of its structure [15,16].

In addition, the CsCRY2 protein domain of *Ch.sichuanensis* determines that it has both light and temperature responses. Previous studies in *Arabidopsis* thaliana have found that *AtCRY2* not only participates in the process of photomorphological construction, but also participates in the regulation of cold resistance under low-temperature stress [17], while *AtCRY1* and *AtCRY3* do not have this function [18,19]. In this study, in the process of exploring *CsCRY2* of *Ch.sichuanensis*, it was found that it also has dual functions of responding to temperature and light (Figure 6). By comparing the changing trend of *CsCRY2* gene expression under different light treatments, it is believed that *CsCRY2* gene may be involved in resisting blue-light stress. Short-term response. In addition, *CsCRY2* was found to have a low-temperature response mechanism through low-temperature stress treatment of *Ch.sichuanensis*. Because the domain of this gene is different from other *CsCRYs* genes (Section 2.1 of this study), it is speculated that this gene is a key member of the *CsCRYs* family in response to light, and it is speculated that CsCRY2, as a blue-light low-temperature cross-signaling node, may regulate downstream stress genes by activating the COP1/HY5 pathway.

From the results of −5 °C treatment, it can be seen that the expression level of CsCRY2 reached its peak here (Figure 6), indicating that it participates in the regulation of cold resistance. Previous studies have shown that Arabidopsis AtCRY2 can integrate light signaling and low-temperature response by activating the COP1/HY5 pathway. The results of this study further support the evolutionary conservation of CsCRY2 as a blue-light low-temperature crossover node.

### 4.2. Subcellular Localization of CsCRys in Chimonobambusa sichuanensis

The results of the subcellular localization of CsCRY1a and CsCRY1b in *Ch.sichuanensis* showed that CsCRY1a and CsCRY1b were located in the cytoplasm, and AtCRY1 in *Arabidopsis thaliana* was a nucleus–cytoplasm transport protein, which could act in both the nucleus and cytoplasm. As a species closely related to *Ch.sichuanensis* in the evolutionary tree, OsCRY1 in rice was also found to play a role in the cytoplasm in previous studies [20]. The subcellular localization of CsCRY2 was found in the nucleus, and AtCRY2 is also a nuclear localization protein in *Arabidopsis*. Both AtCRY3 and CsCRY3 are distributed in chloroplasts, suggesting that they play a role in regulating organelle transcription [21,22,23]. Localization among different species is conservative, which supports the reliability of prediction in this study. In view of the similarity between homologous genes, the similarity rate between CsCRY1a and OsCRY1 (LOC102709715) DNA was 87.98%; the similarity rate between CsCRY1b and OsCRY1 (LOC4336008) DNA was 89.45%; the similarity rate between CsCRY1b and OsCRY1 (LOC102709715) DNA was 89.45%; the similarity between CsCRY2 and OsCRY2 (AB103094.1) was 87.66%; and the similarity rate between CsCRY3 and OsCRY3 (LOC4341749) was 86.16%. Combined with the prediction results of this study, we believe that the cell localization data in rice and *Arabidopsis* can provide indirect support for the localization of CsCRYs, which supports the accuracy of the prediction results. In order to confirm the accuracy of the prediction and build a foundation for subsequent related experiments, it is still necessary to verify the subcellular localization of CsCRYs (see Section 3.5 for the prediction results) and interacting proteins in tobacco through GFP tags in the future, and to construct gene knockout lines to verify the function. The analysis of CsCRYs protein structure by Signalp6.0 software showed that there was no signal peptide structure in the CsCRYs protein of *Ch.sichuanensis*, and its functional domain was mainly responsible for protein binding, which provided help for light signal transmission. This characteristic endows the CsCRYs protein with excellent structural stability and functional diversity. This precise regulation mechanism not only ensures the ion balance between the internal and external environment of the cell, but also is the key to the function of plant photosynthesis.

*Ch.sichuanensis* has multiple ecological advantages such as being evergreen, having cold tolerance, and other characteristics, including special characteristics related to shooting and morphological changes. In this study, based on the previous study demonstrating the blue-light-induced discoloration of *Ch.sichuanensis* bamboo culm, four *CsCRYs* genes were successfully obtained, and their properties and expression under different environmental stresses were further discussed [24].

## Figures and Tables

**Table 1:** Physical and chemical properties and subcellular localization of CsCRYs protein in *Chimonobambusa sichuanensis*.

Indicators	*CsCRY1a*	*CsCRY1b*	*CsCRY2*	*CsCRY3*
Nucleotide length	2139	2094	1476	1833
Protein length	712	697	491	610
Isoelectric point	5.44	5.46	5.08	9.30
Aliphatic index	78.64	80.04	85.58	79.46
Instability index	54.36	51.53	51.19	41.41
Hydrophilic coefficient	−0.433	−0.413	−0.317	−0.356
Relative molecular mass	80.10	79.08	54.15	68.63
α-helix ratio (%)	36.8	39.2	34.6	37.1
Beta-sheet	4.4	4.2	8.4	6.1
Proportion of extended chain (%)	10.7	8.8	10.6	12.5
Subcellular localization	Cytoplasm	Cytoplasm	Nucleus	Chloroplast

**Table 2:** Analysis data for CsCRYs protein three-dimensional prediction diagram and prediction results of the transmembrane structure of the signal peptide.

Indicators	*CsCRY1a*	*CsCRY1b*	*CsCRY2*	*CsCRY3*
Clash score	0.46	1.00	0.51	0.93
Ramachandran outliers	1.00%	1.87%	1.60%	3.62%
Rotamer outliers	1.34%	1.17%	5.05%	2.32%
Cis non-proline	0.77%	0.77%	1.27%	0.18%
Sec/SPI	0	0	0	0
C-score	0.121	0.111	0.115	0.145
S-score	0.149	0.163	0.171	0.388
Y-score	0.124	0.121	0.114	0.153
Mean-S	0.126	0.129	0.117	0.156
Mean-D	0.125	0.125	0.116	0.155
The probability that the N-terminus is on the cytoplasmic side	0.00037	0.00020	0.00370	0.00044
Number of transmembrane amino acids	0.00053	0.00076	0.01276	0.00499
Number of amino acids outside the membrane	712	697	491	610

## Data Availability

The data presented in this study are available on request from the corresponding author. The data will not be disclosed as we have further research to do in our lab.

## Supplementary Materials

The following supporting information can be downloaded at: https://www.mdpi.com/article/10.3390/plants14111637/s1, Figure S1: Predicted transmembrane structure of CsCRYs proteins in *Chimonobambusa sichuanensis*.; Figure S2: Spectra of different light intensity treatment conditions; Table S1: Quantitative primers for the *CsCRYs* in *Chimonobambusa sichuanensis*.
