# Supplementary material for: Identification and Expression Characteristics of the Cryptochrome Gene Family in Chimonobambusa sichuanensis
Source: Plants (Basel). 2025 May 27;14(11):1637. doi: 10.3390/plants14111637 (PMC12157072; doi:10.3390/plants14111637)
Supplement: Supplementary file 1 [file plants-14-01637-s001.zip › plants-3550415-supplementary.pdf]

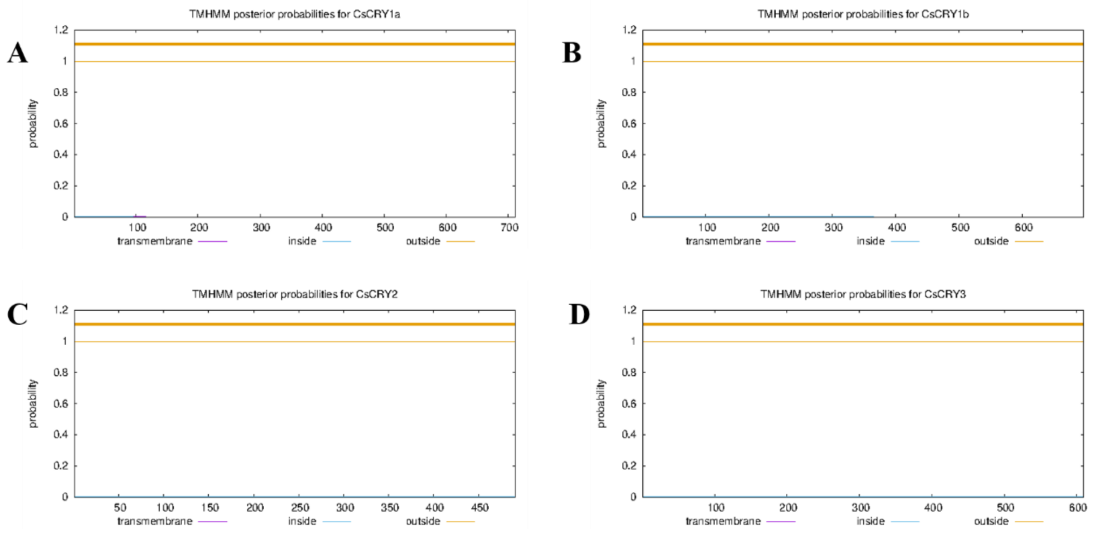

Figure S1 Predicted transmembrane structure of CsCRYs proteins in *Chimonobambusa sichuanensis*.

Note: A: Predicted transmembrane structure of CsCRY1a protein; B: Predicted transmembrane structure of CsCRY1b protein; C: Predicted transmembrane structure of CsCRY2 protein; D: Predicted transmembrane structure of CsCRY3 protein

The quantitative primers are shown in the table

Table S1 Quantitative primers for the *CsCRYs* in *Chimonobambusa sichuanensis*

Gene	F	R
TF-11E	GCAGGTGAAGTTTGGCTGTTATC	CGCCGCTTGGTAGTATTGGT
CsCRY1a	CTTTCAGCGGATTTTGGGA	CGCACACGGTCTAGGCTATTCT
CsCRY1b	GGCATTCACCAGCACAATAACT	GGACCACACCACCTTCTCT
CsCRY2	GTTCTGTCCTGTTCATCTTCAATCG	ACTGGCGTTATCTTCTACCTTACC
CsCRY3	CAACACTGGAGTCACTAGGTCTT	ATGCCGTTCCTGGTCTCTT

And the quantitative amplification procedure is as follows：

Pre - denaturation ----95 ℃,300 s

Denaturation ----95 ℃,30 s
Annealing ----60 ℃,30 s
Extension----72 ℃,30 s
} X35

The quantitative processing conditions are shown in the figure

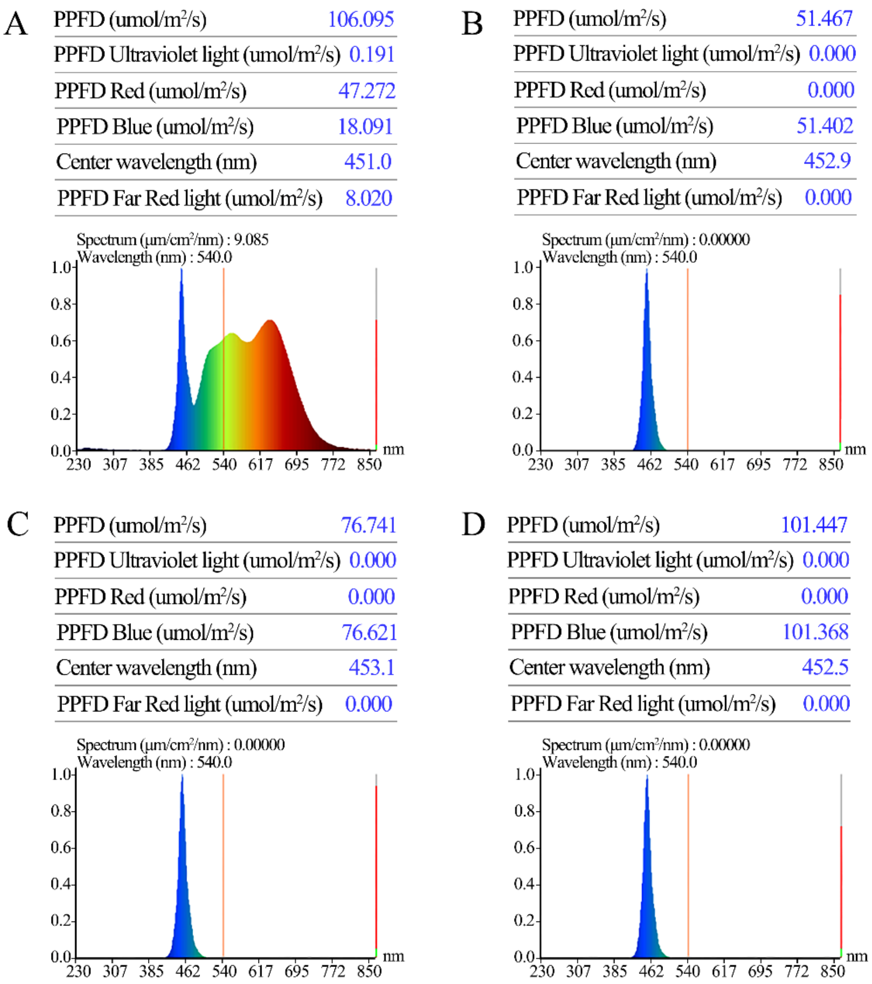

Figure S2 Spectra of different light intensity treatment conditions

Note: A: light intensity spectra of white light treatment group; B: spectrogram with light intensity of 50μmol • $m^{-2}·s^{-1}$; C: spectrogram with light intensity of 75μmol • $m^{-2}·s^{-1}$; D: spectrogram with light intensity of 100μmol • $m^{-2}·s^{-1}$

Note: The original figure is in Chinese, the test parameters have been modified to English figure note
